# Auxiliary roles of nardilysin in the early diagnosis of acute coronary syndrome: a prospective cohort study, the Nardi-ACS study

**DOI:** 10.1007/s11739-023-03508-0

**Published:** 2024-01-17

**Authors:** Mikiko Ohno, Hiroki Shiomi, Osamu Baba, Mariko Yano, Takanori Aizawa, Yukiko Nakano-Matsumura, Shintaro Yamagami, Masashi Kato, Masanobu Ohya, Po-Min Chen, Kazuya Nagao, Kenji Ando, Takafumi Yokomatsu, Kazushige Kadota, Ichiro Kouchi, Tsukasa Inada, Cindy Valentine, Takahiro Kitagawa, Masato Kurokawa, Shigeru Ohtsuru, Takeshi Morimoto, Takeshi Kimura, Eiichiro Nishi

**Affiliations:** 1 Department of Pharmacology, Shiga University of Medical Science, Seta Tsukinowa-Cho, Otsu, Shiga 520-2192 Japan; 2 Department of Cardiovascular Medicine, Graduate School of Medicine, Kyoto University, 54 Shogoin-Kawahara-Cho, Sakyo-Ku, Kyoto, 606-8507 Japan; 3 Preemptive Medicine and Lifestyle Disease Research Center, Kyoto University Hospital, 54 Shogoinkawahara-Cho, Sakyo-Ku, Kyoto, 606-8507 Japan; 4 Kokura Memorial Hospital, 3-2-1 Asano, Kita-Ku, Kokura, Kitakyushu, Fukuoka 802-8555 Japan; 5 Mitsubishi Kyoto Hospital, 1, Katsuragoshomachi, Nishikyo-Ku, Kyoto, 615-8087 Japan; 6 Kurashiki Central Hospital, 1-1-1 Miwa, Kurashiki, Okayama 710-8602 Japan; 7 Saiseikai Noe Hospital, 1-3-25, Furuichi, Joto-Ku, Osaka 536-0001 Japan; 8 Osaka Red-Cross Hospital, 5-30 Fudegasakicho, Tennoji-Ku, Osaka 543-8555 Japan; 9 Sanyo Chemical Industries, 11-1 Hitotsubashi Nomoto, Higashiyama, Kyoto, 605-0995 Japan; 10 Department of Primary Care and Emergency Medicine, Graduate School of Medicine, Kyoto University, 54 Shogoinkawahara-Cho, Sakyo-Ku, Kyoto, 606-8507 Japan; 11 Hyogo College of Medicine, 1-1, Mukogawa-Cho, Nishinomiya, Hyogo 663-8501 Japan; 12 Present Address: Hirakata Kosai Hospital, 1-2-1, Fujibanto-Cho, Hirakata, Osaka 573-0153 Japan

**Keywords:** Acute coronary syndrome, Biomarker, Early diagnosis, Nardilysin, Cardiac troponin

## Abstract

Acute coronary syndrome (ACS) includes myocardial infarction (MI) and unstable angina (UA). MI is defined by elevated necrosis markers, preferably high-sensitivity cardiac troponins (hs-cTn). However, it takes hours for cTn to become elevated after coronary occlusion; therefore, difficulties are associated with diagnosing early post-onset MI or UA. The aim of this prospective cohort study was to examine the diagnostic ability of serum nardilysin (NRDC) for the early detection of ACS. This study consisted of two sequential cohorts, the Phase I cohort, 435 patients presenting to the emergency room (ER) with chest pain, and the Phase II cohort, 486 patients with chest pain who underwent coronary angiography. The final diagnosis was ACS in 155 out of 435 patients (35.6%) in the phase I and 418 out of 486 (86.0%) in the phase II cohort. Among 680 patients who presented within 24 h of onset, 466 patients (68.5%) were diagnosed with ACS. Serum NRDC levels were significantly higher in patients with ACS than in those without ACS. The sensitivity of NRDC in patients who presented within 6 h after the onset was higher than that of hsTnI, and the AUC of NRDC within 1 h of the onset was higher than that of hsTnI (0.718 versus 0.633). Among hsTnI-negative patients (300 of 680 patients: 44.1%), 136 of whom (45.3%) were diagnosed with ACS, the sensitivity and the NPV of NRDC were 73.5 and 65.7%, respectively. When measured in combination with hsTnI, NRDC plays auxiliary roles in the early diagnosis of ACS.

**Supplementary Information:**

The online version contains supplementary material available at 10.1007/s11739-023-03508-0.

## Introduction

Acute coronary syndrome (ACS), the leading cause of death worldwide, consists of ST elevation myocardial infarction (STEMI) and non-ST elevation ACS (NSTE-ACS). NSTE-ACS is further divided into NSTE-MI and unstable angina (UA) by the presence or absence of myocardial injury, which is defined by an increase in a necrosis marker, preferably cardiac troponins (cTn). Since cTn, the structural proteins of the myocardium, are expressed almost exclusively in the heart, elevated cTn values specifically reflect injury to cardiomyocytes. However, since it takes hours for cardiomyocyte necrosis after coronary occlusion in human, elevation of cTn is sometimes undetectable in the early post-onset patients with MI [1]. Difficulties are associated with the diagnosis of early post-onset MI, particularly if its clinical presentation, including symptoms and electrocardiogram, is atypical.

High-sensitivity (hs)–cTn assays have enabled the detection of even small myocardial injuries, and this has resulted in an increase in the diagnosis of MI and a reciprocal decrease in that of UA [2, 3]. Although the risk of death is lower among patients with UA than among those with MI patients, it is important to correctly diagnose UA because a substantial proportion of UA patients will develop MI [1]. There are currently no biomarkers for transient severe ischemia in UA.

ACS is characterized by atherosclerotic plaque rupture and intraluminal thrombosis. Chronic inflammation and the resulting tissue damage underlie the pathology of atherosclerosis, in which innate and adaptive immune systems play important roles [4]. We previously screened autoantibodies using serum from ACS patients and identified 19 candidate antigens including nardilysin (*N*-arginine dibasic convertase; NRDC) [5]. NRDC is a metalloendopeptidase of the M16 family, which is expressed in a wide range of organs including the heart. NRDC shows a unique pattern of cellular localization in the cytosol, mitochondria, and nucleus, and is exported out of cells via an unconventional secretory pathway. In the extracellular space, NRDC enhances the activity of a dis-integrin and metalloproteinases (ADAM) to release an ectodomain of membrane proteins such as tumor necrosis factor alpha (TNF-α), while NRDC in the nucleus coregulates transcription with transcription factor partners [6, 7]. Analyses of the gene-manipulated mice revealed the critical in vivo roles of NRDC in several biological processes and the pathology of inflammatory diseases and cancers [8–18]. We established a highly sensitive measurement system for NRDC that enables the detection of serum NRDC, and applied it to human studies [15, 16]. We previously demonstrated that serum NRDC levels were significantly higher in patients with than in those without ACS [5]. Furthermore, NRDC was elevated in patients with UA in whom the necrosis marker creatine kinase (CK) was negative [5]. Since the positive rate of NRDC was very high at the admission of ACS patients, NRDC has potential as a marker for the very early detection of ACS [5].

Our previous clinical study was a single-center retrospective cohort study; therefore, we herein conducted a multicenter prospective cohort study to examine the actual value of serum NRDC measurements for the early detection of ACS. In the Phase I primary cohort, we consecutively enrolled patients with chest pain at the emergency room (ER) to confirm the clinical performance of NRDC testing. In the Phase II cohort, which sequentially followed the primary cohort, we have enrolled patients with chest pain who underwent cardiac catheterization or coronary computed tomography (CT) angiography to specifically focus on the patients with ACS early after its onset.

## Methods

### Study design and participants

**Fig. 1:**
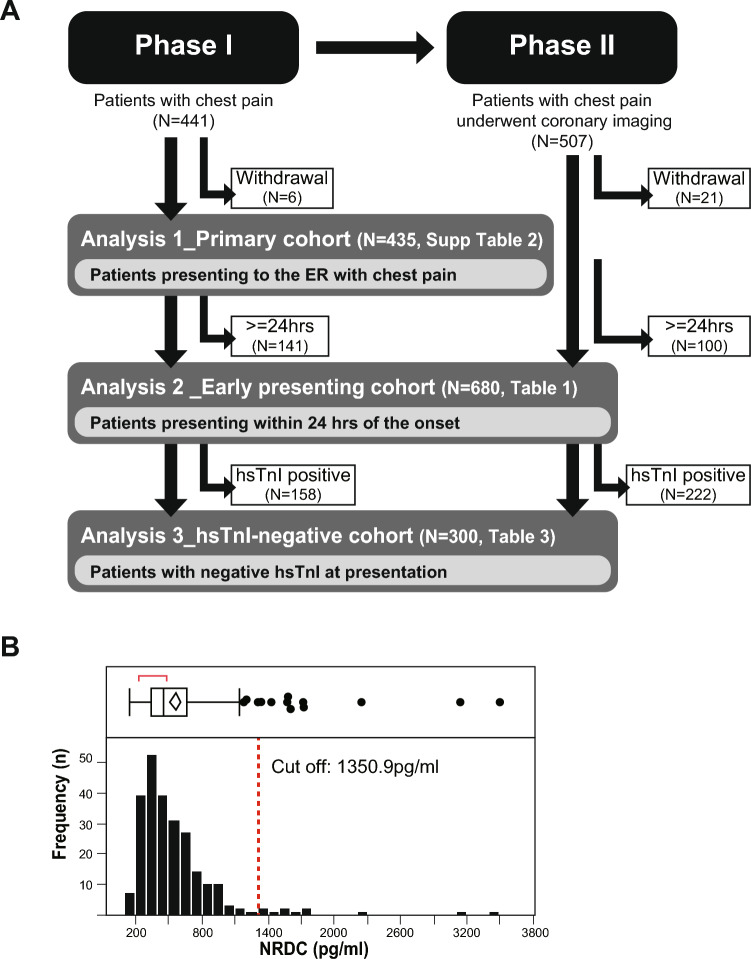
**A** Flow diagram of the process of enrollment, exclusion and analysis. **B** Distribution of serum NRDC values in the healthy volunteers (*N* = 246)

The prospective evaluation of Nardilysin for the early detection of patients with Acute Coronary Syndrome (Nardi-ACS study) was conducted as a multicenter, prospective, observational, cohort study in six hospitals in Japan (Kyoto University Hospital, Kokura Memorial Hospital, Mitsubishi Kyoto Hospital, Kurashiki Central Hospital, Saiseikai Noe Hospital, and Osaka Red Cross Hospital). The present study conformed to the principles outlined in the Declaration of Helsinki and the study protocol was approved by the Ethics Committee of each participating institution. Written informed consent was obtained from each participant. This study consisted of two sequential Phase I and II cohorts. In the Phase I cohort, we prospectively and consecutively enrolled 441 patients with chest pain at the ER to evaluate the diagnostic ability of NRDC for ACS in a general ER setting. We analyzed 435 patients because six patients were excluded because blood was not collected or due to a request to withdraw (Analysis 1: Fig. 1A, Supplementary Table 2). In the Phase II cohort, we prospectively and consecutively enrolled 507 patients with chest pain at the ER who were suspected of having ACS and underwent cardiac catheterization or coronary CT angiography, in which 21 patients were excluded because blood was not collected or due to a request to withdraw. Since the major aim of the present study was to evaluate the clinical value of NRDC in the early detection of ACS, patients in the Phase I and II cohorts with known times of onset were stratified by the time from the onset of chest pain to blood collection, and 680 patients who presented the ER within 24 h of the onset were separately analyzed (Early presenting cohort, Analysis 2: Fig. 1A, Table 1). Of 680 patients, 300 patients with negative hsTnI at initial blood test were separately analyzed (Analysis 3: Fig. 1A, Table 3). Patient enrollment periods differed among the hospitals, details of which are as follows: November 2016 to August 2017 for Phase I and August 2017 to January 2020 for Phase II in Kyoto University Hospital, December 2016 to February 2017 for Phase I and February 2017 to October 2019 for Phase II in Kokura Memorial Hospital, February 2017 to August 2017 for Phase I and September 2017 to March 2020 for Phase II in Mitsubishi Kyoto Hospital, January 2017 to February 2017 for Phase I and November 2018 to February 2019 for Phase II in Kurashiki Central Hospital, May 2017 to June 2017 for Phase I and June 2017 to May 2018 for Phase II in Saiseikai Noe Hospital, May 2018 to September 2018 for Phase II Osaka Red Cross Hospital. This study was registered in UMIN Clinical Trials Registry as UMIN000024577.

**Table 1:** Patient characteristics of 680 patients who presented within 24 h of symptom onset (phase I & II cohorts)

*N* = 680	ACS		Non-ACS		
*N*	466	68.5%	214	31.5%	
Phase I	121	41.2%	173	58.8%	
Phase II	345	89.4%	41	10.6%	(*P*)
Age (mean ± SD)	70.4	12.5	70.8	15.8	0.7042
Gender (male: *N*, %)	356	52.4%	127	18.7%	<0.0001
BMI (mean ± SD)	23.6	3.82	23.1	4.04	0.2117
STEMI (*N*, %)	340	50.0%			
NSTE-ACS (*N*, %)	126	18.5%			
Hs troponin-I-negative (*N*, %)	136	29.2%	164	76.6%	
STEMI (*N*, % of ACS)	96	20.6%			
NSTE-ACS (*N*, % of ACS)	40	8.6%			
UA (*N*, % of ACS)	29	6.2%			

<Past history>	N	%	N	%	(*P*)
Diabetes mellitus	173	25.4	52	7.7	0.0011
Hypertension	309	45.4	125	18.4	0.1234
OMI	63	9.3	34	5.0	0.6873
PCI	92	13.5	54	7.9	0.1742
CABG	15	2.2	9	1.3	0.4185
Vascular disease	66	9.7	25	3.7	0.617
Dyslipidemia	223	32.8	86	12.7	0.0342
Current smoking	137	20.2	32	4.7	<0.0001
Hemodialysis	18	2.7	8	1.2	0.9818
Chronic kidney disease (eGFR < 30)	46	6.8	15	2.2	0.4731
Malignancy	51	7.5	20	2.9	0.3645

<Laboratory data>	Mean	SD	Mean	SD	(*P*)
WBC/μL	9190.7	3066.0	7148.8	2666.1	<0.0001
Hemoglobin, g/dL	13.8	2.1	13.2	2.0	0.0006
Platelets ×10^4^/μL	22.1	6.9	22.3	9.1	0.8176
AST, IU/L	58.1	75.3	36.0	54.1	0.0001
ALT, IU/L	32.7	38.2	25.9	26.1	0.0183
LDH, IU/L	291.3	184.4	225.9	71.6	<0.0001
CK, IU/L	408.5	733.8	159.9	283.9	<0.0001
CK-MB, IU/L	34.8	67.9	12.7	44.0	<0.0001
Peak CK,IU/L	2004.1	2267.0	202.6	585.0	<0.0001
Peak CK-MB, IU/L	192.1	222.8	17.2	99.5	<0.0001
Serum creatinine, mg/dL	1.21	1.55	1.10	1.21	0.3509
BUN, mg/dL	19.4	10.0	18.5	13.5	0.2817
Uric acid, mg/dL	5.7	2.2	5.5	1.5	0.1304
Total cholesterol, mg/dL	190.9	47.7	184.4	41.3	0.1099
HDL-cholesterol, mg/dL	48.4	12.9	55.1	16.2	<0.0001
LDL-cholesterol, mg/dL	118.3	37.9	104.3	31.4	<0.0001
Triglyceride, mg/dL	143.9	104.1	137.6	87.3	0.4984
C-reactive protein, mg/dL	0.90	2.27	0.63	1.57	0.1295
Hemoglobin A1C, %	6.5	1.30	6.1	0.91	0.0045
BNP, pg/mL	195.5	408.3	213.4	451.1	0.6705
NRDC, pg/mL	4009.4	4614.0	2325.0	2142.7	<0.0001
hsTnI, pg/mL	11,596.9	37,060	522.1	2152.8	<0.0001

*BMI* body mass index, *STEMI* *ST elevation* myocardial infarction, *NSTE-ACS* non-*ST* elevation acute coronary syndrome, *UA* unstable angina, *OMI* old myocardial infarction, *PCI* percutaneous coronary intervention, *CABG* coronary artery bypass graft, *WBC* white blood cell, *AST* aspartate aminotransferase, *ALT* alanine aminotransferase, *LDH* lactate dehydrogenase, *CK* creatine kinase, *CK-MB* creatine kinase-muscle/brain, *BUN* blood urea nitrogen, *HDL* high-density lipoprotein, *LDL* low-density lipoprotein, *BNP* brain natriuretic peptide

### Diagnosis of ACS

ACS, including STEMI, NSTEMI, and UA was defined according to the standard criteria as previously described [1, 19, 20]. The diagnosis of MI was based on the universal definition of MI (third and fourth Universal Definition 2012 & 2018). UA was diagnosed in patients with symptoms of myocardial ischemia, which was judged by the attending physicians, and the severe stenosis (> 90%) of a major coronary artery in the absence of cardiomyocyte necrosis (no elevations in CK, CK-MB, and hsTn). In-house hsTn was measured by the laboratory at each institution and reflected in actual clinical decisions. hsTnI values used in the present study were measured in batches at the core laboratory of Abbott Japan using the ARCHITECT STAT High Sensitive Troponin-I assay kit. The cut-off level for hsTnI was set at 52 pg/mL according to the 0 h/1 h rule-in and rule-out algorithms described in the 2015 European Society of Cardiology (ESC) guideline for the management of ACS in patients presenting without persistent ST-segment elevation [20].

### Data and blood sample collection

Clinical and laboratory parameters at baseline were collected from medical records at each hospital. Venous blood samples were obtained from patients at the ER and placed in test tubes containing polyolefin resin (Terumo). Serum was separated by centrifugation and stored at −80 ℃ for later analyses. In patients diagnosed with acute myocardial infarction (AMI), additional serum sampling was performed every 4 h (Kurashiki Central Hospital) or 6 h (the other five hospitals) after admission until peak CPK or CK-MB levels were reached, which is routinely conducted according to the local rules of coronary care units. Serum hsTnI and NRDC levels were measured at the core laboratories of Abbott Japan and Sanyo Chemical Industries, Ltd., respectively.

### Measurement of serum NRDC

Serum NRDC was quantified by a chemiluminescent enzyme immunoassay as previously described, in which mouse monoclonal anti-NRDC antibody clones #231 and #304 were used for the capture and detection antibodies, respectively [5, 15]. In the present study, NRDC in all samples was measured using an automated chemiluminescent immune assay analyzer, the Accuraseed system (FUJIFILM Wako Pure Chemical Corporation). Briefly, 25 μL of serum was mixed with the capture antibody (clone #231) immobilized to magnetic particles (MAGRAPID^®^) and incubated at 37 ℃ for 180 s. After washing the magnetic particles, the detection antibody (#304) conjugated with peroxidase was added and incubated at 37 ℃ for 180 s. Magnetic particles were aggregated by a magnet and the solution was removed. After washing the magnetic particles, they were dispersed by removing the magnet, and then 200 μL of the substrate solution was then added to measure chemiluminescence. The assay for serum NRDC was calibrated using human recombinant NRDC, synthesized using a silkworm protein expression system (Sysmex), as reference material.

To determine the diagnostic cut-off level in the new Accuraseed system, NRDC was measured in 246 healthy individuals (169 males and 77 females, mean age; 48.8 ± 9.35), who were selected from 1466 individuals who underwent medical check-ups at the Preemptive Medicine and Lifestyle Disease Research Center in Kyoto University Hospital between July 2019 and June 2020. Exclusion criteria were as follows: (1) those receiving treatment for any chronic disease at the time of the medical check-up; (2) those in whom new diseases requiring treatment were identified in the medical check-up; (3) those whose blood tests showed any abnormality in the medical check-up. The mean and standard deviations (SD) of serum NRDC in the 246 healthy individuals selected were 561.9 pg/mL and 394.5 pg/mL, respectively. We defined the cut-off value as the mean + 2SD, 1350.9 pg/mL (Fig. 1B), which was close to the previous cut-off level (1418.3 pg/mL) determined by the old measurement system using the SphereLight 180 analyzer (Olympus) [5]. The one-way layout of serum NRDC in each final diagnosis (Phases I & II) is described in Supplementary Fig. A.

### Follow-up

The incidence of major cardiac adverse events (MACE), the components of which were defined as new lethal or non-lethal MI, sudden death, and heart failure, in all participants was analyzed for 30 days from the time of the first blood sample collection. The 30-day follow-up of MACE was performed by the attending physician through the medical records or contact by phone calls.

### Statistical analysis

Continuous variables are presented as the mean ± SD or standard error (SE) as described, or the median with the interquartile range, and were compared using the Student’s *t*-test (two groups) or an analysis of variance (ANOVA) (*n* > 2 groups) with Turkey’s post hoc tests. Categorical variables were presented as numbers with percentages and were compared using the *χ*^2^ test. To evaluate the test performance of serum NRDC and hsTnI as diagnostic markers for ACS, the area under the curve (AUC) of the receiver operating characteristics curve (ROC) was calculated. Additionally, sensitivity, specificity, positive predictive values (PPV), and negative predictive values (NPV) for the target markers were assessed by the defined marker-specific cut-off value and a 2 × 2 table in the usual manner. All statistical analyses were performed using the JMP version 10 statistical package.

## Results

### Baseline characteristics

The Phase I cohort included 435 patients with chest pain at the ER, with 155 being diagnosed with ACS (36%). The Phase II cohort consisted of 486 patients with chest pain who were highly suspected of having ACS and underwent cardiac catheterization or coronary CT angiography, and 418 patients were finally diagnosed with ACS (86%) (Fig. 1A and Supplementary Table 1). Among the 921 patients analyzed in the Phase I & II cohort, 680 visited the ER within 24 h after the onset of chest pain (Early presenting cohort). Of the 680 patients in the Early presenting cohort, 300 patients had negative hsTnI on the initial blood test (hsTnI-negative cohort). The baseline characteristics of patients in the Phase I and the Early presenting cohort are shown in Supplementary Table 2 and Table 1, respectively.

### Diagnostic ability of serum NRDC in a general ER setting (Analysis 1)

In the Phase I primary cohort, serum NRDC levels in the initial blood test at the ER were significantly higher in patients with than in those without ACS (2639.0 ± 3115.9 pg/mL versus 2113.2 ± 2200.2 pg/mL, *P* = 0.041; Supplementary Table 2). The sensitivity, specificity, PPV, and NPV of NRDC for ACS were 63.2, 47.9, 51.3, and 70.2 (%), respectively. The ROC analysis showed that the AUC of NRDC was 0.5834, while that of hsTnI was 0.7966 (Supplementary Fig. B, C).

### Diagnostic ability of serum NRDC in patients presented early after the onset (Analysis 2)

**Fig. 2:**
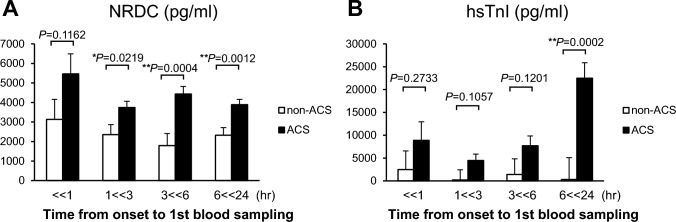
**A**, **B** Average values of NRDC and hsTnI in patients with ACS or non-ACS whose blood was collected at the indicated time period (h) from the onset of chest pain. Data are shown as mean ± standard error (SE). *P < 0.05, **P < 0.01

Since the major aim of the present study was to evaluate the clinical value of NRDC in the early detection of ACS, patients in the Phase I and II cohorts with known times of onset were stratified by the time from the onset of chest pain to blood collection. As described in Table 1, blood was collected from 680 (466 ACS and 214 non-ACS) patients within 24 h of the onset, with the highest number of patients presenting within 1–3 h of the onset. In these patients, serum NRDC levels were higher in patients with than in those without ACS (4009.4 ± 4614.0 pg/mL versus 2325.0 ± 2142.7 pg/mL, *P* < 0.001; Table 1). Similar results were observed when the patients were divided into the following groups: within 1 h, between 1 and 3 h, between 3 and 6 h, and between 6 and 24 h after the onset (Fig. 2A). In contrast, there were no significant differences in hsTnI levels between ACS and non-ACS patients in the groups within 6 h, while hsTnI levels were markedly higher in patients with ACS from 6 to 24 h after the onset (Fig. 2B). Consistent with this result, the sensitivity of NRDC for the diagnosis of ACS within 6 h after the onset was higher than that of hsTnI (0.955 versus 0.409 within 1 h, 0.746 versus 0.529 between 1 and 3 h, 0.851 versus 0.819 between 3 and 6 h; Table 2), whereas the specificity of NRDC was markedly lower than that of hsTnI. The AUC of NRDC within 1 h of the onset was also higher than that of hsTnI (0.718 versus 0.633).

**Table 2:** Diagnostic performance of NRDC (left) and hsTnI (right) for ACS in patients who presented ER within 24 h of onset

NRDC							hsTnI						
Time from onset (h)	*N*	Sensitivity	Specificity	PPV	NPV	AUC	Time from onset (h)	*N*	Sensitivity	Specificity	PPV	NPV	AUC
≪ 1	22	0.955	0.227	0.553	0.833	0.718	≪ 1	22	0.409	0.727	0.600	0.552	0.633
1 ≪ 3	189	0.746	0.351	0.746	0.351	0.592	1 ≪ 3	189	0.529	0.797	0.870	0.399	0.746
3 ≪ 6	94	0.851	0.432	0.792	0.533	0.745	3 ≪ 6	94	0.819	0.730	0.885	0.614	0.777
6 ≪ 24	161	0.752	0.407	0.716	0.452	0.638	6 ≪ 24	161	0.894	0.765	0.883	0.785	0.913
Total	466	0.779	0.374	0.730	0.437	0.643	Total	466	0.708	0.766	0.868	0.547	0.803

### Diagnostic ability of serum NRDC in hsTnI-negative patients (Analysis 3)

We then focused on hsTnI-negative patients. Among 680 patients who presented within 24 h of the onset, 300 patients (44.1%) were negative for hsTnI at the initial blood draw, 136 of whom (45.3%) were ultimately diagnosed with ACS. Among hsTnI-negative patients, the sensitivity, specificity, PPV, and NPV of NRDC for ACS were 73.5, 42.1, 51.3, and 65.7 (%), respectively. The AUC of NRDC in these patients was 0.608 (Table 3). When divided into four groups by the time from the onset of chest pain onset to blood collection, the AUC of NRDC was 0.712 within 1 h, 0.567 between 1 and 3 h, 0.736 between 3 and 6 h, and 0.516 between 6 and 24 h (Table 3). These results indicate that the diagnostic capacity of NRDC for ACS remained constant regardless of positive or negative hsTnI.

**Table 3:** Diagnostic performance of NRDC for ACS in hsTnI-negative patients

Time from onset	Sensitivity	Specificity	PPV	NPV	AUC	ACS (*N*)	Non-ACS (*N*)	Total (*N*)^a^
≪ 1	0.923	0.250	0.500	0.800	0.712	13	16	29
1 ≪ 3	0.708	0.373	0.630	0.458	0.567	89	59	148
3 ≪ 6	0.824	0.481	0.500	0.813	0.736	17	27	44
6 ≪ 24	0.647	0.484	0.256	0.833	0.516	17	62	79
Total	0.735	0.421	0.513	0.657	0.608	136	164	300

^a^hsTnI-negative ACS and hsTnI-negative non-ACS

In ACS, the diagnosis of NSTE-ACS including UA in hsTnI-negative patients is particularly challenging. Therefore, we analyzed the AUC of NRDC (NSTE-ACS versus non-ACS in hsTnI-negative patients), which was 0.499 in total (0.781 within 1 h, 0.437 between 1 and 3 h, 0.672 between 3 and 6 h, and 0.573 between 6 and 24 h) (Supplementary Table 3). The sensitivity, specificity, PPV, and NPV of NRDC for NSTE-ACS in hsTnI-negative patients were 62.5, 42.1, 20.8, and 82.1 (%), and for UA were 58.6, 42.1, 15.2, and 85.2 (%), respectively (Supplementary Tables 3, 4).

### Time course of serum NRDC in patients with ACS

**Fig. 3:**
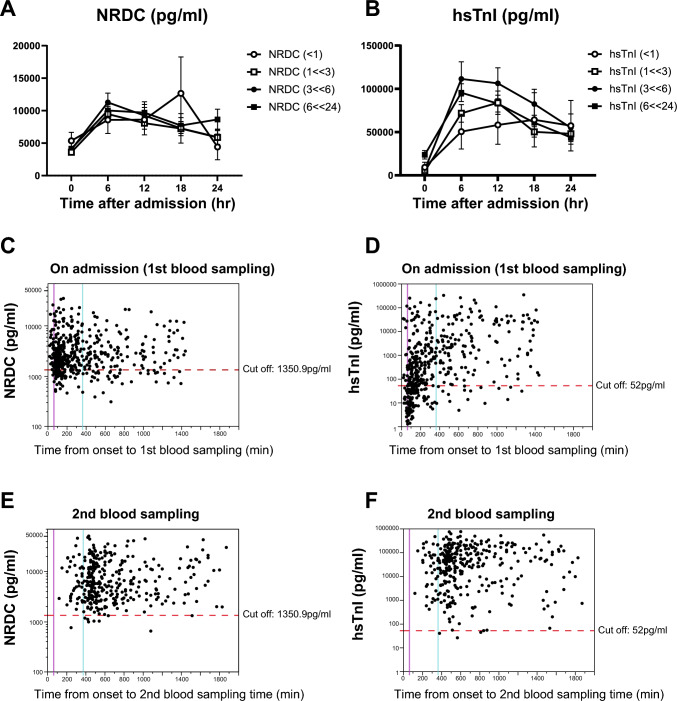
**A**, **B** Time course analysis of NRDC (**A**) and hsTnI (**B**) in ACS patients who required hospitalization. Blood tests were performed at admission (Time 0) and every 6 h after the admission. Time after the onset of chest pain to the initial blood sampling is within 1 h (open circle), between 1 and 3 h (open square), between 3 and 6 h (closed circle), and between 6 and 24 h (closed square). **C**–**F** NRDC (**C**, **E**) and hsTnI (**D**, **F**) values at the first (**C**, **D**) and second (**E**, **F**) blood sampling were blotted with the time from the onset of chest pain to the blood sampling

A serial blood test until CK peaks is routinely performed in coronary care units in Japan to estimate the extent of the infarcted region. To assess the dynamic state in blood, NRDC and hsTnI were measured in ASC patients on admission and every 6 h after the admission (Fig. 3A, B). NRDC and hsTnI values at the first and second blood sampling were also blotted with the time from the onset of chest pain to the blood collection (Fig. 3C–F). For hsTnI, the shorter the time since onset, the lower the value tended to be (Fig. 3B, D), whereas NRDC was already high at the time on admission regardless of the time since onset (Fig. 3A, C). In the second blood sampling, almost all patients have an increase in both NRDC and hsTnI (Fig. 3E, F).

### Thirty days MACE

We also estimated MACE, defined as the composite of new MI, sudden death, and heart failure, by following up patients for 30 days after the initial blood test. Among 881 (847 + 34) patients in the phase I and II cohorts (follow-up rate of 95.7%), 34 (3.7%) developed MACE (Supplementary Table 5). Serum NRDC levels in the initial blood test were significantly higher in MACE-positive patients compared with MACE-negative patients (5344.1 ± 3780.9 pg/mL versus 3192.5 ± 3780.8 pg/mL, *P* = 0.0012; Supplementary Table 3). hsTnI levels were also significantly higher in MACE-positive patients (18,945.1 ± 26,933.7 pg/mL versus 6677.6 ± 26,935.1 pg/mL, *P* = 0.0094).

## Discussion

This prospective study was conducted to evaluate the diagnostic performance of NRDC in ACS. Our previous retrospective cohort study provided two important findings: (1) serum NRDC was elevated in the very early phase of ACS, and (2) serum NRDC was elevated not only in patients with AMI, but also in those with UA [5]. These clinical findings suggested that NRDC is a biomarker with a pathophysiological background independent of cell necrosis. This hypothesis was also supported by cell-based experiments, in which the short treatment of rat cardiomyocytes with H_2_O_2_ or A23187 induced the secretion of NRDC without cell toxicity [5].

To verify whether the measurement of serum NRDC is useful for the early diagnosis of ACS, we have prospectively enrolled patients with chest pain at the ER. In the Phase I primary cohort, we consecutively enrolled all patients with chest pain to evaluate the general diagnostic power of NRDC. In the Phase II cohort, we enrolled patients who presented to the ER with chest pain and underwent cardiac catheterization or coronary CT angiography. As a result, 36% of patients in the Phase I cohort and 86% in the Phase II cohort were finally diagnosed with ACS. When restricted to patients who presented within 24 h of the onset, the percentage of ACS patients was 41% in the Phase I cohort and 89% in the Phase II cohort, indicating that the change in the enrollment process resulted in the 2.2-fold more efficient registration of early post-onset ACS patients. One possible reason for the high percentages of ACS both in the Phase I and II cohorts is that two out of the six participating hospitals are high-volume centers for ACS.

The AUC of NRDC in the Phase I primary cohort (0.5834) was lower than that reported in the previous study (0.822) [5]. This may have been due to control cases in the previous study including patients with arrhythmia or stable angina who were admitted for standby procedures, whereas control cases in the present study were non-ACS patients presenting to the ER with chest pain. In early post-onset ACS patients (within 24 h), serum NRDC levels were higher in patients with than in those without ACS. The AUC of NRDC in patients presenting within 1 h of the onset was higher than that of cTnI. In patients presenting between 1 and 3 h and between 3 and 6 h, serum NRDC levels, but not cTnI levels were significantly higher in ACS patients than in non-ACS patients. These results indicate that serum NRDC is useful for the early diagnosis of ACS, whereas the specificity of NRDC was lower than that of hsTnI. NRDC is expressed in a wide range of organs and cells and is involved in inflammatory diseases. The present results demonstrated that serum NRDC levels were also elevated in some patients with non-ACS cardiovascular diseases (CVD), such as acute pericarditis, Takotsubo syndrome (TTS), and congestive heart failure (CHF), and also in those with non-CVD, such as pneumonia, which may account for low specificity in the diagnosis of ACS. In consistent, serum NRDC was significantly correlated with WBC, CRP, and several necrosis markers (LDH, peak CK) (Supplementary Table 6), suggesting that serum NRDC increased with the intensity of inflammation and myocardial damage. NRDC may also be useful in terms of the triage of patients requiring hospitalization.

Since ACS is a life-threatening disease, its rapid and accurate diagnosis is critical. At the same time, the ability to rule out ACS safely and promptly is highly valuable in terms of saving medical resources. Regarding triage, ESC proposed the 0 h/1 h rule-out and rule-in algorithm using the high-sensitivity cardiac troponins (hs-cTn) assay [21]. Several large cohort studies showed that the NPV for MI in patients assigned ‘rule-out’ exceeded 99%, while the PPV for MI in patients assigned ‘rule-in’ was approximately 70–75% [3, 22]. However, one of the issues with this algorithm is that a large percentage of patients do not qualify for rule-out or rule-in and, thus are assigned to the ‘observe’ zone. In this study, blood samples were not taken 1 h after the visit; therefore, if the 0 h algorithm in 2020 ESC guidelines [21] was applied, 243 out of 680 patients (36%) who presented within 24 h of the onset were assigned to the ‘observe’ zone (4 < hsTnI < 64) by the initial blood sampling. Among these patients, 137 patients (56%) were finally diagnosed with ACS. The simultaneous measurement of NRDC at presentation may have assisted the quick rule-in and rule-out by the PPV of 62.0% and NPV of 55.0%. Although this diagnostic capability is not perfect, but it will facilitate a diagnosis under conditions where cTn is negative. The number of patients in the ‘observe’ zone has been reported to increase with age, which decreases the efficacy of the triage [23]. It would be interesting to examine if the diagnostic ability of NRDC is affected by age or not.

In this study, sensitivity and specificity of hsTnI for ACS in early presenting cohort are 71% and 77%, respectively. The relatively low sensitivity can be attributed to the high cutoff value at 52 ng/L, which was set according to the rule-in level of the 0 h algorithms described in the 2015 ESC guideline. We set this high cutoff because we would like to test the use of serum NRDC with hsTnI for the quick rule-in. The low sensitivity can be also attributed to the fact that there were many patients who presented to the ER very early after the onset (< 3 h).

The mechanisms by which NRDC increases very early after the onset and its significance in the pathogenesis of ACS currently remain unclear. Given the role of NRDC in the activation of TNF-alpha, which was validated in some inflammatory disease models including rheumatoid arthritis and steatohepatitis [13, 24], NRDC may aggravate the pathology of ACS by enhancing inflammation. Another possible involvement of NRDC in ACS pathology is through the production of platelet [25, 26], because NRDC secretion is induced by turbulent flow, which promotes thrombopoiesis in vitro [27]. Based on these findings, a novel therapy targeting NRDC may inhibit the progression of ACS by suppressing inflammation and thrombosis.

## Limitation

There are several limitations that need to be addressed. The present study was conducted on patients who presented to the ER with chest pain. Further studies are needed to examine the utility of NRDC in patients with a lower probability of ACS in a general practitioner setting. Although we used a stringent method to adjudicate the presence or absence of ACS, including central measurements of hs-cTn and invasive or non-invasive coronary artery imaging, we still may have misclassified a small number of patients. The cut-off level of hsTnI was set at 52 pg/mL according to the 0 h rule-in and rule-out algorithms described in the 2015 ESC guidelines, which has since changed to 64 pg/mL in the revised 2020 ESC guidelines [21]. As the detailed echocardiographic data were not included in this study, the relationship between serum NRDC and left ventricular function or valvular diseases could not be addressed. Since we did not perform specific sample size calculations, the present study may have been underpowered for some comparisons.

## Conclusions

The present study demonstrated the potential of NRDC as a biomarker for the early detection of ACS. The AUC of NRDC for the diagnosis of ACS in patients who presented within 1 h after the onset was higher than that of hsTnI. When we focused on hsTnI-negative patients, the sensitivity, specificity, PPV, and NPV of NRDC for ACS were 73.5, 42.1, 51.3, and 65.7 (%). These results suggest auxiliary roles for NRDC in the early diagnosis of ACS.

### Supplementary Information

Below is the link to the electronic supplementary material.

Supplementary file1 (PDF 2405 KB)

## Data Availability

The datasets generated and/or analyzed in the present study are not publicly available due to policy issues in the hospitals but are available from the corresponding authors on reasonable request.

## Abbreviations

ACS: Acute coronary syndrome
MI: Myocardial infarction
UA: Unstable angina
hs-cTn: High-sensitivity cardiac troponins
NRDC: Nardilysin
ER: Emergency room
AUC: Area under the curve
PPV: Positive predictive value
NPV: Negative predictive value
STEMI: ST elevation myocardial infarction
NSTE-ACS: Non-ST elevation ACS
ADAM: A disintegrin and metalloproteinases
TNF-alpha: Tumor necrosis factor alpha
CK: Creatine kinase
CT: Computed tomography
CK-MB: Creatine kinase-muscle/brain
SD: Standard deviation
MACE: Major cardiac adverse events
SE: Standard error
ANOVA: Analysis of variance
ROC: Receiver operating characteristics curve
CCU: Critical care unit
AMI: Acute myocardial infarction
CVD: Cardiovascular diseases
TTS: Takotsubo syndrome
CHF: Congestive heart failure
ESC: European Society of Cardiology

## References

[1] Thygesen K, Alpert JS, Jaffe AS, Chaitman BR, Bax JJ, Morrow DA, White HD, Executive Group on behalf of the Joint European Society of Cardiology/American College of Cardiology/American Heart Association/World Heart Federation Task Force for the Universal Definition of Myocardial Infarction (2018). Fourth universal definition of myocardial infarction (2018). Circulation.

[2] Shah AS, Anand A, Sandoval Y, Lee KK, Smith SW, Adamson PD, Chapman AR, Langdon T, Sandeman D, Vaswani A (2015). High-sensitivity cardiac troponin I at presentation in patients with suspected acute coronary syndrome: a cohort study. Lancet.

[3] Boeddinghaus J, Twerenbold R, Nestelberger T, Badertscher P, Wildi K, Puelacher C, du Fay de Lavallaz J, Keser E, Rubini Gimenez M, Wussler D (2018). Clinical validation of a novel high-sensitivity cardiac troponin I assay for early diagnosis of acute myocardial infarction. Clin Chem.

[4] Meier LA, Binstadt BA (2018). The contribution of autoantibodies to inflammatory cardiovascular pathology. Front Immunol.

[5] Chen PM, Ohno M, Hiwasa T, Nishi K, Saijo S, Sakamoto J, Morita Y, Matsuda S, Watanabe S, Kuwabara Y (2017). Nardilysin is a promising biomarker for the early diagnosis of acute coronary syndrome. Int J Cardiol.

[6] Nishi E, Rawlings ND, Salvesen G (2013). Nardilysin. Handbook of proteolytic enzymes.

[7] Nishi E, Prat A, Hospital V, Elenius K, Klagsbrun M (2001). N-arginine dibasic convertase is a specific receptor for heparin-binding EGF-like growth factor that mediates cell migration. EMBO J.

[8] Ohno M, Hiraoka Y, Matsuoka T, Tomimoto H, Takao K, Miyakawa T, Oshima N, Kiyonari H, Kimura T, Kita T (2009). Nardilysin regulates axonal maturation and myelination in the central and peripheral nervous system. Nat Neurosci.

[9] Hiraoka Y, Matsuoka T, Ohno M, Nakamura K, Saijo S, Matsumura S, Nishi K, Sakamoto J, Chen PM, Inoue K (2014). Critical roles of nardilysin in the maintenance of body temperature homoeostasis. Nat Commun.

[10] Ohno M, Hiraoka Y, Lichtenthaler SF, Nishi K, Saijo S, Matsuoka T, Tomimoto H, Araki W, Takahashi R, Kita T (2014). Nardilysin prevents amyloid plaque formation by enhancing alpha-secretase activity in an Alzheimer’s disease mouse model. Neurobiol Aging.

[11] Nishi K, Sato Y, Ohno M, Hiraoka Y, Saijo S, Sakamoto J, Chen PM, Morita Y, Matsuda S, Iwasaki K (2016). Nardilysin is required for maintaining pancreatic beta-cell function. Diabetes.

[12] Ohno M, Nishi K, Hiraoka Y, Niizuma S, Matsuda S, Iwasaki H, Kimura T, Nishi E (2020). Nardilysin controls cardiac sympathetic innervation patterning through regulation of p75 neurotrophin receptor. FASEB J.

[13] Fujii T, Nishi E, Ito H, Yoshitomi H, Furu M, Okabe N, Ohno M, Nishi K, Morita Y, Morita Y (2017). Nardilysin is involved in autoimmune arthritis via the regulation of tumour necrosis factor alpha secretion. RMD Open.

[14] Kanda K, Sakamoto J, Matsumoto Y, Ikuta K, Goto N, Morita Y, Ohno M, Nishi K, Eto K, Kimura Y (2018). Nardilysin controls intestinal tumorigenesis through HDAC1/p53-dependent transcriptional regulation. JCI Insight.

[15] Kanda K, Komekado H, Sawabu T, Ishizu S, Nakanishi Y, Nakatsuji M, Akitake-Kawano R, Ohno M, Hiraoka Y, Kawada M (2012). Nardilysin and ADAM proteases promote gastric cancer cell growth by activating intrinsic cytokine signalling via enhanced ectodomain shedding of TNF-alpha. EMBO Mol Med.

[16] Kasai Y, Toriguchi K, Hatano E, Nishi K, Ohno M, Yoh T, Fukuyama K, Nishio T, Okuno M, Iwaisako K (2017). Nardilysin promotes hepatocellular carcinoma through activation of signal transducer and activator of transcription 3. Cancer Sci.

[17] Kimura Y, Ikuta K, Kimura T, Chiba T, Oshima H, Oshima M, Nishi E, Seno H (2017). Nardilysin regulates inflammation, metaplasia, and tumors in murine stomach. Sci Rep.

[18] Yoh T, Hatano E, Kasai Y, Fuji H, Nishi K, Toriguchi K, Sueoka H, Ohno M, Seo S, Iwaisako K (2019). Serum nardilysin, a surrogate marker for epithelial-mesenchymal transition, predicts prognosis of intrahepatic cholangiocarcinoma after surgical resection. Clin Cancer Res.

[19] Thygesen K, Alpert JS, Jaffe AS, Simoons ML, Chaitman BR, White HD, Katus HA, Lindahl B, Morrow DA, Joint ESCAAHAWHFTFftUDoMI (2012). Third universal definition of myocardial infarction. Circulation.

[20] Roffi M, Patrono C, Collet JP, Mueller C, Valgimigli M, Andreotti F, Bax JJ, Borger MA, Brotons C, Chew DP (2016). 2015 ESC guidelines for the management of acute coronary syndromes in patients presenting without persistent ST-segment elevation: task force for the management of acute coronary syndromes in patients presenting without persistent ST-segment elevation of the European Society of Cardiology (ESC). Eur Heart J.

[21] Collet JP, Thiele H, Barbato E, Barthelemy O, Bauersachs J, Bhatt DL, Dendale P, Dorobantu M, Edvardsen T, Folliguet T (2021). 2020 ESC guidelines for the management of acute coronary syndromes in patients presenting without persistent ST-segment elevation. Eur Heart J.

[22] Twerenbold R, Badertscher P, Boeddinghaus J, Nestelberger T, Wildi K, Puelacher C, Sabti Z, Rubini Gimenez M, Tschirky S, du Fay de Lavallaz J (2018). 0/1-Hour triage algorithm for myocardial infarction in patients with renal dysfunction. Circulation.

[23] Boeddinghaus J, Nestelberger T, Twerenbold R, Neumann JT, Lindahl B, Giannitsis E, Sorensen NA, Badertscher P, Jann JE, Wussler D (2018). Impact of age on the performance of the ESC 0/1h-algorithms for early diagnosis of myocardial infarction. Eur Heart J.

[24] Ishizu-Higashi S, Seno H, Nishi E, Matsumoto Y, Ikuta K, Tsuda M, Kimura Y, Takada Y, Kimura Y, Nakanishi Y (2014). Deletion of nardilysin prevents the development of steatohepatitis and liver fibrotic changes. PLoS ONE.

[25] Ly HQ, Kirtane AJ, Murphy SA, Buros J, Cannon CP, Braunwald E, Gibson CM, Group TS (2006). Association of platelet counts on presentation and clinical outcomes in ST-elevation myocardial infarction (from the TIMI Trials). Am J Cardiol.

[26] Wu Y, Wu H, Mueller C, Gibson CM, Murphy S, Shi Y, Xu G, Yang J (2012). Baseline platelet count and clinical outcome in acute coronary syndrome. Circ J.

[27] Ito Y, Nakamura S, Sugimoto N, Shigemori T, Kato Y, Ohno M, Sakuma S, Ito K, Kumon H, Hirose H et al (2018) Turbulence activates platelet biogenesis to enable clinical scale ex vivo production. Cell 174(3):636–648.e618 10.1016/j.cell.2018.06.011 30017246

