# Supplementary material for: Auxiliary roles of nardilysin in the early diagnosis of acute coronary syndrome: a prospective cohort study, the Nardi-ACS study
Source: Intern Emerg Med. 2024 Jan 17;19(3):649–59. doi: 10.1007/s11739-023-03508-0 (PMC11039555; doi:10.1007/s11739-023-03508-0)
Supplement: Supplementary file 1 — Supplementary file1 (PDF 2405 KB) [file 11739_2023_3508_MOESM1_ESM.pdf]

Supplementary Figure

A One-way layout of serum NRDC in each diagnosis (Phase I & II cohorts)

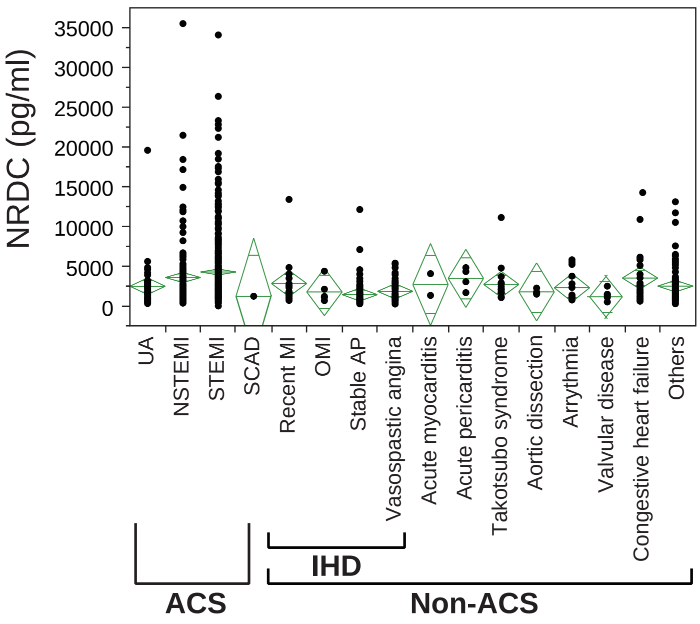

B

ROC (NRDC)

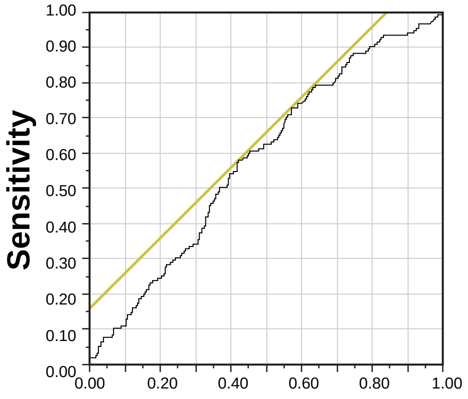

1-Specificity

AUC 0.5834

C

ROC (hsTnI)

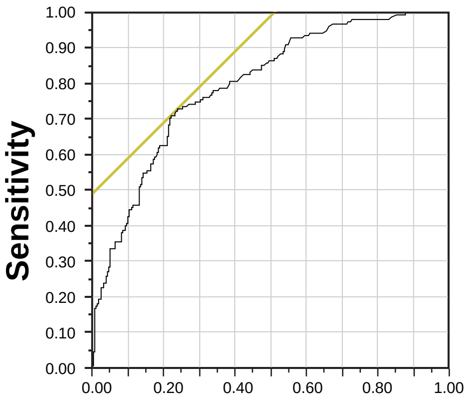

1-Specificity

AUC 0.7966

A: One-way layout of serum NRDC in each diagnosis (Phase I and Phase II cohorts).
Patient numbers for each diagnosis are shown in Supplementary Table 1.
Abbereviations: UA: unstable angina, NSTEMI: non ST elevation myocardial infarction, STEMI: ST elevation myocardial infarction, SCAD: spontaneous coronary dissection, OMI: old myocardial infarction, AP: angina pectoris, Others: Non-cardiovascular diseases
B, C: Diagnostic power of NRDC and hsTnI for ACS in the Phase I cohort.
Receiver operating characteristic (ROC) curves and corresponging area under the curve (AUC) of NRDC (B) and hsTnI (C) are shown.

Supplementary Table 1 :
Patient characteristics and final diagnosis in Phase I and II cohorts.

	All			
	Phase I	Phase II	Total	
**Number**	**435**	**486**	**921**	***p***
**Age (mean +/- sd)**	70.5 +/- 14.7	70.4 +/- 12.5	70.5 +/- 13.6	0.940
**Gender (male: N, %)**	286 (65.7%)	343 (70.6%)	629 (68.3%)	0.119
**BMI (mean +/- sd)**	23.2 +/- 4.0	23.6 +/- 3.7	23.4 +/- 3.9	0.132
**Diabetes Mellitus**	129	179	308	0.021
**Hypertension**	256	331	587	0.003
**OMI (in past history)**	68	60	128	0.182
**Vascular disease**	48	68	116	0.164
**Dyslipidemia**	173	241	414	0.002
**Current smoking**	70	140	210	<0.0001
**Hemodialysis**	19	20	39	1.000
**Chronic kidney disease (eGFR<30)**	34	52	86	0.140
**Malignancy**	51	47	98	0.669
**ACS: Number (%)**	**155 (35.6%)**	**418 (86%)**	**573 (62.2%)**	
**CAG in ACS: N (%)**	**155 (100%)**	**418 (100%)**	**573 (100%)**	
**NSTE-ACS**	57	125	182	
**UA**	29	45	74	
**NSTEMI**	28	80	108	
**(with CPA)**	1	0	1	
**STEMI**	98	293	391	
**(with CPA)**	2	6	8	
**(with shock)**	0	4	4	
**(with Vf)**	0	2	2	
**(SCAD)**	0	1	1	
**Non-ACS: Number (%)**	**280 (64.4%)**	**68 (14.0%)**	**348 (37.8%)**	
**CAG in non-ACS: N (%)**	**83 (29.6%)**	**62 (91.1%)**	**145 (41.7%)**	
**Recent MI**	6	14	20	
**Stable AP**	79	3	82	
**OMI**	6	0	6	
**VSA**	43	15	58	
**Acute myocarditis**	1	1	2	
**Acute pericarditis**	2	2	4	
**Takotsubo syndrome**	4	16	20	
**Aortic dissection**	4	0	4	
**Arrythmia**	14	3	17	
**Valvular disease**	6	1	7	
**CHF**	19	9	28	
**Others**	96	4	100	

Abbreviations: BMI: body mass index, OMI: old myocardial infarction, ACS: acute coronary syndrome, CAG: coronary angiography

NSTE-ACS: non ST elevation-acute coronary syndrome, STEMI: ST-elevation myocardial infarction, CPA: cardiopulmonary arrest

Vf: Ventricular fibrillation, SCAD: spontaneous coronary artery dissection, AP: angina pectoris, VSA: vasospastic angina, CHF: congestive heart failure

Supplementary Table 2 : Patient characteristics of 435 patients in Phase I cohort.

	ACS		Non-ACS			Phase I	
N	155	35.6%	280	64.4%	(P)	435	100%
Age (mean+/-sd)	70.8	11.7	70.3	16.2	0.75	70.5	14.7
Sex (male: N, %)	120	27.6%	166	38.2%	0.0001	286	65.7%
BMI (mean+/-sd)	23.4	4.07	23.1	4.00	0.38	23.2	4.03
hsTnI negative (n, %)	61	39.4%	228	81.4%			
<Past history>	N	%	N	%	(P)	N	%
Diabetes Mellitus	53	12.2	76	17.5	0.29	129	29.7
Hypertension	95	21.8	161	37.0	0.51	256	58.9
OMI	25	5.7	43	9.9	0.75	68	15.6
PCI	41	9.4	67	15.4	0.44	108	24.8
CABG	6	1.4	10	2.3	0.61	16	3.7
Vascular disease	19	4.4	29	6.7	0.33	48	11.0
Dyslipidemia	72	16.6	101	23.2	0.10	173	39.8
Current smoking	35	8.0	35	8.0	0.01	70	16.1
Hemodialysis	4	0.9	15	3.4	N.A.	19	4.4
Chronic kidney disease	9	2.1	25	5.7	0.24	34	7.8
Malignancy	23	5.3	28	6.4	0.22	51	11.7
<Laboratory data>	Mean	sd	Mean	sd	(P)	Mean	sd
WBC/μL	8417.7	3050.0	7240.4	5060.0	0.0087	7660.0	4480.0
Hemoglobin, g/dL	13.7	2.16	13.0	2.02	0.0016	13.3	2.09
Platelets $\times 10^3$/μL	21.8	6.61	21.9	7.62	0.9040	21.9	7.27
AST, IU/L	51.8	61.4	34.8	51.2	0.0022	40.9	55.6
ALT, IU/L	27.1	17.9	25.7	26.7	0.5698	26.2	23.9
LDH, IU/L	275.6	170.0	223.8	88.9	<.0001	242.3	126.4
CK, IU/L	392.3	608.0	174.8	411.1	<.0001	257.3	505.5
CK-MB, IU/L	36.5	60.3	12.2	41.8	<.0001	21.6	51.1
Serum creatinine, mg/dL	1.08	1.13	1.22	1.50	0.3032	1.17	1.38
BUN, mg/dl	19.3	10.2	19.0	10.2	0.737	19.1	10.2
Uric acid, mg/dL	5.7	1.46	5.5	1.58	0.1136	5.6	1.54
Total cholesterol, mg/dL	192.1	42.8	182.3	38.0	0.0207	185.9	40.1
HDL-cholesterol, mg/dL	50.7	13.5	53.7	15.6	0.0678	52.5	14.8
LDL-cholesterol, mg/dL	117.8	36.7	104.0	29.5	0.0002	109.5	33.2
Triglyceride, mg/dL	133.9	97.6	136.9	89.0	0.7699	135.7	92.4
C-reactive protein, mg/dL	0.73	2.01	1.06	3.55	0.2851	0.94	3.08
Hemoglobin A1C, %	6.48	1.27	6.18	0.88	0.0116	6.3	1.06
BNP, pg/mL	174.3	356.1	223.0	430.1	0.3244	205.2	404.7
NRDC, pg/ml	2639.0	3115.9	2113.2	2200.2	0.0411	2300.6	2573.0
hsTnI, pg/ml	9713.9	33695.8	1080.2	10562.1	<.0001	4156.6	22175.2

Abbreviations: BMI: body mass index, OMI: old myocardial infarction, PCI: percutaneous coronary intervention, CABG: coronary artery bypass graft, WBC: white blood cell, AST: Aspartate Aminotransferase, ALT: Alanine Aminotransferase, LDH: Lactate Dehydrogenase, CK: Creatine Kinase, CK-MB: Creatine Kinase-Muscle/Brain, BUN: Blood Urea Nitrogen, HDL: High-Density Lipoprotein, LDL: Low-Density Lipoprotein, BNP: Brain Natriuretic Peptide

Supplementary Table 3 :

Diagnostic performance of NRDC for NSTE-ACS in hsTnI-negative patients

Time after onset (hr)	Sensitivity	Specificity	PPV	NPV	AUC	NSTE-ACS (N)	Total (N)
<<1	1.000	0.250	0.143	1.000	0.781	2	18
1<<3	0.600	0.373	0.245	0.733	0.437	20	79
3<<6	0.714	0.481	0.263	0.867	0.672	7	34
6<<24	0.545	0.484	0.158	0.857	0.573	11	73
Total	0.625	0.421	0.208	0.821	0.499	40	204

Supplementary Table 4 :

Diagnostic performance of NRDC for UA in hsTnI-negative patients.

Time after onset (hr)	Sensitivity	Specificity	PPV	NPV	AUC	UA (N)	Total (N)
<<1	N.A.	0.250	0.000	1.000	N.A.	0	16
1<<3	0.583	0.373	0.159	0.815	0.422	12	71
3<<6	0.714	0.481	0.263	0.867	0.672	7	34
6<<24	0.500	0.484	0.135	0.857	0.579	10	72
Total	0.586	0.421	0.152	0.852	0.476	29	193

Supplementary Table 5 :

Incidence of 30-day MACE in all patients and mean value of NRDC and hsTnI in patients with or without MACE.

	MACE (-)	MACE (+)	
**Number (%)**	847 (92%)	34 (3.7%)	*p*
**NRDC** mean +/- SD (pg/ml)	3192.5 +/- 3780.8	5344.1 +/- 3780.9	0.0012
**hsTnI** mean +/- SD (pg/ml)	6677.6 +/- 26935.1	18945.1 +/- 26933.7	0.0094

Abbreviations: MACE: Major Adverse Cardiac Events, SD: standard deviation

Supplementary Table 6 :

Correlation between NRDC and other biomarkers

All			ACS			non-ACS		
**Risk profiles or laboratory data**	**Spearman's rank correlation coefficients (ρ)**	**Prob> |ρ|**	**Risk profiles or laboratory data**	**Spearman's rank correlation coefficients (ρ)**	**Prob> |ρ|**	**Risk profiles or laboratory data**	**Spearman's rank correlation coefficients (ρ)**	**Prob> |ρ|**
WBC	0.4004	<.0001	WBC	0.3125	<.0001	WBC	0.3838	<.0001
LDH	0.3409	<.0001	LDH	0.2846	<.0001	LDH	0.3761	<.0001
peak CK-MB	0.315	<.0001	AST	0.2401	<.0001	peak CK-MB	0.3663	<.0001
AST	0.2956	<.0001	CRP	0.2166	<.0001	AST	0.2576	<.0001
peak CPK	0.2621	<.0001	peak CK-MB	0.1987	<.0001	CRP	0.2299	<.0001
CRP	0.2351	<.0001	ALT	0.198	<.0001	Cre	0.198	0.0002
ALT	0.2229	<.0001	peak CPK	0.1926	<.0001	ALT	0.1957	0.0003
BS	0.1438	<.0001	Hb	0.0979	0.0192	BNP	0.1799	0.0052
Hb	0.123	0.0002	plt	0.0722	0.0986	BS	0.1768	0.002
Cre	0.1073	0.0011	BS	0.0495	0.2514	BUN	0.1457	0.0067
BNP	0.1016	0.0104	BNP	0.0372	0.4611	UA	0.1116	0.0457
UA	0.0791	0.021	UA	0.0303	0.4869	peak CPK	0.1023	0.1356
HbA1c	0.0523	0.1571	Cre	0.0161	0.7015	HbA1c	0.0808	0.1978
BUN	0.044	0.1834	HbA1c	-0.0078	0.8647	Hb	0.0372	0.4906
plt	0.0431	0.2048	BUN	-0.0237	0.5721	TG	0.0125	0.8393
LDL-cho	0.0203	0.5806	LDL-cho	-0.0237	0.6057	plt	-0.0069	0.8988
TG	-0.047	0.198	T-cho	-0.0681	0.1216	LDL-cho	-0.0241	0.6961
T-cho	-0.0501	0.1516	TG	-0.0983	0.0306	T-cho	-0.0703	0.2213
HDL-cho	-0.1765	<.0001	HDL-cho	-0.1423	0.0019	HDL-cho	-0.0981	0.1119

Abbreviations: WBC: White Blood Cell, LDH: Lactate Dehydrogenase, peak CK-MB: peak Creatine Kinase-Muscle/ Brain, AST: Aspartate Aminotransferase, peak CPK: peak Creatine Phosphokinase, CRP: C-reactive Protein, ALT: Alanine Aminotransferase, BS: Blood Sugar, Hb: Hemoglobin, Cre: Creatinine, BNP: Brain Natriuretic Peptide, UA: Uric Acid, HbA1c: Hemoglobin A1c, BUN: Blood Urea Nitrogen, plt: Platelet, LDL-cho: Low-Density Lipoprotein Cholesterol, TG: Triglycerides, T-cho: Total Cholesterol, High-Density Lipoprotein Cholesterol
